# Suicidal ideation among patients with facial pain disorders

**DOI:** 10.1097/PR9.0000000000001415

**Published:** 2026-05-13

**Authors:** Justin M. Campbell, Rose Caston, Omid Shoraka, John D. Rolston, Shervin Rahimpour

**Affiliations:** a Spencer Fox Eccles School of Medicine, University of Utah, Salt Lake City, UT, USA; b Department of Neurosurgery, University of Wisconsin-Madison, Madison, WI, USA; c Department of Neurosurgery, Clinical Neuroscience Center, University of Utah, Salt Lake City, UT, USA; d Department of Neurosurgery, Mass General Brigham, Harvard Medical School, Boston, MA, USA; e Department of Biomedical Engineering, University of Utah, Salt Lake City, UT, USA

**Keywords:** Facial pain, Trigeminal neuralgia, Suicide

## Abstract

**Introduction:**

Facial pain can be severely debilitating. Although etiologies vary, facial pain commonly results in diminished quality of life and a pronounced psychosocial burden.

**Objectives:**

To determine whether severe facial pain is associated with a heightened risk of suicidal ideation.

**Methods:**

We performed a retrospective review of patients with facial pain disorders who, during a presurgical evaluation, completed the Columbia-Suicide Severity Rating Scale and Brief Pain Inventory-Facial (BPI-Facial) scale, which assess suicidal risk and the effect of facial pain on quality of life, respectively. To evaluate the independent association between components of the BPI-Facial scale and suicidal thoughts or behaviors, we constructed a series of multivariable logistic regression models. Further classification and k-means clustering techniques were used to predict individual instances of suicidal ideation and to group distinct phenotypes of facial pain, respectively.

**Results:**

We identified 181 patients with facial pain who met the inclusion criteria. The most common cause of facial pain was trigeminal neuralgia (78.5%). Within our cohort, 13.3% of patients reported some degree of suicidal ideation in the Columbia-Suicide Severity Rating Scale questionnaire, a rate approximately 3 times higher than the estimated prevalence in the general adult population. This heightened risk of suicide was associated with considerable pain-related impairments reported in the BPI-Facial scale across the following tested domains: pain intensity, interference in general activities, and interference in face-specific activities.

**Conclusion:**

Our results suggest that facial pain can have highly detrimental effects on patient well-being. Moreover, quantifying facial pain-related impairments may assist in developing targeted interventions and longitudinal screening for mitigating the heightened risk of suicide among those with facial pain disorders.

## Abstract 2

Patients with facial pain disorders exhibited a heightened risk of suicide that was associated with considerable pain-related impairments.

## 1. Introduction

Facial pain disorders, although relatively rare, can result in severe pain that significantly impairs a patient's quality of life.^9^ The etiologies and clinical presentations of facial pain are varied—facial pain may be caused by neurovascular conflict (eg, classical trigeminal neuralgia), occur secondary to other medical comorbidities (eg, multiple sclerosis), or be idiopathic.^18^ Trigeminal neuralgia (TN), a condition characterized by excruciating paroxysmal pain in the dermatomal distribution of the trigeminal nerve, is one of the most common and debilitating facial pain disorders.^4,8,11^

Severe facial pain often entails a considerable psychosocial burden stemming from high rates of disability^21^ and numerous psychiatric comorbidities.^6,19,20^ Moreover, patients with TN and other severe facial pain disorders exhibit high levels of pain catastrophizing,^12^ which can exacerbate feelings of helplessness.^14^ Indeed, TN has sometimes been termed “the suicide disease” because of the unbearable pain.

Although chronic nonfacial pain has been associated with a heightened risk of suicide,^2,7,15^ the prevalence of suicidal ideation in those with facial pain disorders is poorly characterized. To address this knowledge gap, we analyzed a large cohort of patients with facial pain disorders who completed a screening questionnaire for suicidal ideation and a multidimensional assessment of the effect of facial pain on their activities of daily living. We hypothesized that, in association with the self-reported impairments to their quality of life, patients with facial pain disorders would exhibit a higher rate of suicidal ideation compared with the general population.

## 2. Methods

### 2.1. Data collection and curation

We performed a retrospective review of medical records of patients treated at our institution that contained a diagnostic (International Classification of Diseases) code for facial pain. Demographic data and clinical characteristics (eg, cause of facial pain, symptom onset) were collected. The onset of symptoms was determined by reviewing clinical notes; dates were approximated using available information in patient records. During their initial visit, patients were asked to complete 2 clinical questionnaires, which are described below. This retrospective study was approved by our Institutional Review Board with a waiver of patient consent.

### 2.2. Clinical questionnaires

#### 2.2.1. Columbia-Suicide Severity Rating Scale

The Columbia-Suicide Severity Rating Scale (C-SSRS) is a brief screening questionnaire for suicidal risk that assesses the presence of active or passive suicidal thoughts (eg, wish to be dead) and suicidal behavior (eg, suicide attempts) in patients.^13^ The questionnaire consists of 6 possible questions with “yes”/“no” answers (eg, “Have you done anything, started to do anything, or prepared to do anything to end your life?”). The responses to each question are used to stratify suicide risk (ie, low, moderate, or high) and determine whether immediate support is necessary. In the version used for this study, the first 5 items assess suicidal ideation within the past month, whereas the final item pertains to preparatory or attempt behaviors occurring at any point in one's lifetime or within the past 3 months. Because later items are administered only if the patient endorses recent suicidal thoughts, all patients identified as exhibiting suicidal ideation in our analyses, by definition, reported thoughts of self-harm within the month before assessment.

#### 2.2.2. Brief Pain inventory-Facial Scale

The Brief Pain Inventory-Facial (BPI-Facial) scale measures impairments across 3 distinct domains: pain intensity (eg, average level of pain or pain at its worst), interference in general activities (eg, mood or sleep), and interference in face-specific activities (eg, talking or eating).^10,17^ There are 18 items, each of which is scored on an 11-point scale ranging from 0 (ie, “No pain,” “Does not interfere”) to 10 (ie, “Pain as bad as you can imagine,” “Completely interferes”). The BPI-Facial has demonstrated strong internal consistency (overall Cronbach α = 0.94), construct validity supported by factor analysis, and expert clinical review supporting its content and face validity.^10^

### 2.3. Statistical analysis

Patient demographics and clinical characteristics were summarized using the appropriate descriptive statistics. We simplified the analysis of the clinical questionnaires in 2 ways. First, responses to the C-SSRS were binarized such that patients who responded “yes” to any of the screening questions were labeled as having suicidal ideation. Second, we used the mean score across items within each of the 3 domains of the BPI-Facial scale. We used 2 × 2 chi-square tests for independence to contrast the proportion of patients with suicidal ideation across potential predictors (eg, sex, facial pain cause) and Welch t-tests to compare BPI-Facial scale domain scores between groups because the observed variance was heterogeneous (Levene test, *P* < 0.05).

Associations between exposure variables and binary outcomes were examined using univariable and multivariable logistic regression analyses. Multivariable models were adjusted for potential confounders, including age, sex, and race. Model discrimination was assessed using receiver operating characteristic (ROC) curve analysis, with the area under the curve (AUC) calculated to quantify each model's ability to distinguish between outcome groups. For continuous predictors, Youden index (YI) was calculated from the ROC curve to identify the optimal cut-off point that maximized the sum of sensitivity and specificity for predicting the binary outcome. All statistical analyses were performed using custom Python scripts, SPSS (IBM Corp), and R (R Foundation for Statistical Computing).

As an exploratory analysis, we examined whether particular ranges of BPI-Facial scores most effectively discriminated between individuals with and without suicidal ideation. To this end, we applied machine-learning techniques to perform distinct but complementary prediction-based analyses. First, we developed a logistic regression–based classifier to predict individual instances of suicidal ideation in unseen data. Next, we implemented an exploratory k-means clustering approach to determine whether patients could be grouped into distinct phenotypes of facial pain. For both techniques, we used the same set of 6 features: sex, facial pain diagnosis (for simplicity, TN or not TN), age, and the mean scores across the pain intensity, interference in general activities, and interference in face-specific activities domains.

To train the classifier, we used fivefold cross-validation (80/20 train/test split) and a synthetic minority oversampling technique to mitigate the effect of imbalanced classes.^3^ Classifier performance was evaluated by calculating the ROC-AUC and by examining the model coefficients, which provided insights into which features were most influential. Clusters identified using the k-means approach were visualized using the first 2 principal components.

## 3. Results

**Figure 1.**
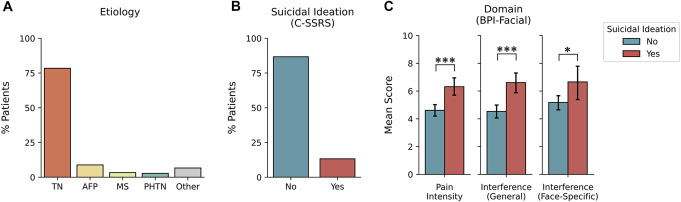
Summary of facial pain cause and responses to clinical questionnaires. (A) Comparison of the number of patients with different etiologies of facial pain. TN, trigeminal neuralgia; AFP, atypical facial pain; MS, multiple sclerosis; PHTN, postherpetic trigeminal neuralgia. (B) Responses from the Columbia-Suicide Severity Rating Scale (C-SSRS), where “yes” indicates ≥1 response confirming suicidal thoughts or behaviors. (C) Mean scores (0 indicates no impairment, 10 indicates considerable impairment) on the Brief Pain Inventory-Facial (BPI-Facial) questionnaire within the 3 domains (pain intensity, interference in general activities, and interference in face-specific activity), split by C-SSRS response. Error bars represent the 95% confidence interval. *P < 0.05, **P < 0.01, ***P < 0.001.

We identified 181 patients with facial pain diagnoses who underwent presurgical evaluation at our institution between July 2020 and September 2024 and met our inclusion criteria. The mean (±SD) age of the cohort was 58.4 (±18.2) years, 68.5% were female, and 84.0% were White. The most common cause of facial pain was classical TN (78.5%), followed by atypical facial pain (8.8%), multiple sclerosis (3.3%), and postherpetic TN (2.8%); 6.6% of patients had facial pain that was otherwise unspecified (labeled “Other”) (Fig. 1A). The median (Q1, Q3) time between the earliest mention of facial pain disorder in the medical record and the index patient encounter was approximately 6.3 years (2.9, 12.3).

Analysis of C-SSRS responses revealed that 13.3% of patients exhibited some degree of suicidal ideation or suicidal behaviors (Fig. 1B). Patients also reported considerable pain-related impairment across each of the domains tested with the BPI-Facial scale (mean ± SD): pain intensity (4.8 ± 2.6), interference in general activities (4.8 ± 2.9), and interference in face-specific activities (5.4 ± 3.5). Patients who exhibited suicidal ideation reported higher scores in the pain intensity (6.3 ± 1.6 vs 4.6 ± 2.7; *t*(175) = 4.27, *P* < 0.001), interference in general activities (6.6 ± 1.8 vs 4.5 ± 3.0; *t*(175) = 4.66, *P* < 0.001), and interference in face-specific activities domains (6.7 ± 2.9 vs 5.2 ± 3.5; *t*(175) = 2.16, *P* = 0.038) (Fig. 1C). We did not observe a difference in the proportions of patients with suicidal ideation when grouping patients by sex (*χ*^2^(1) = 2.08, *P* = 0.15) or whether they were diagnosed with TN (*χ*^2^(1) = 0.31, *P* = 0.86).

To evaluate the independent association between components of the BPI-Facial scale and suicidal thoughts or behaviors, 4 separate inferential multivariable logistic regression models were constructed. Each model included one of the following BPI-Facial scale domain mean scores as the primary predictor: (1) interference with general activities, (2) interference with face-specific activities, (3) pain intensity, or (4) the total BPI-Facial scale score. Age, race, and sex were included in all models as covariates to adjust for potential confounding. In adjusted analyses, higher scores for interference with general activities (odds ratio [OR] = 1.34, 95% confidence interval [95% CI] = 1.09 to 1.63, *P* = 0.005), pain intensity (OR = 1.37, 95% CI = 1.09–1.71, *P* = 0.006), and the total BPI-Facial scale score (OR = 1.01, 95% CI = 1.00–1.03, *P* = 0.008) were significantly associated with a higher odds of exhibiting suicidal thoughts or behaviors (Table 1). Interference with face-specific activities showed a nonsignificant association (OR = 1.14, 95% CI = 0.99–1.32, *P* = 0.066).

**Table 1:** Univariable and multivariable logistic regression models examining the association between each domain of the Brief Pain Inventory-Facial scale and suicidal thoughts or behaviors.

Independent variable	Univariable		Multivariable		Multivariable model AUC
	OR/β (95% CI)	*P*	OR/β (95% CI)	*P*	
Pain interference with general activity	1.35 (1.12–1.64)	**0.002**	1.34 (1.09–1.63)	**0.005**	0.776
Pain interference with facial activity	1.16 (1.01–1.33)	**0.039**	1.14 (0.99–1.32)	0.066	0.725
Pain severity	1.33 (1.09–1.62)	**0.005**	1.37 (1.09–1.71)	**0.006**	0.777
Total BPI-Facial scale score	1.02 (1.00–1.03)	**0.004**	1.01 (1.00–1.03)	**0.008**	0.770

Separate multivariable models were developed for each predictor. Each multivariable model was adjusted for age, race, and sex, in addition to the independent variable listed in the corresponding row.

Boldface font indicates statistical significance at *P* < 0.05.

AUC, area under the curve; BPI-Facial, Brief Pain Inventory-Facial; CI, confidence interval; OR, odds ratio.

**Figure 2.**
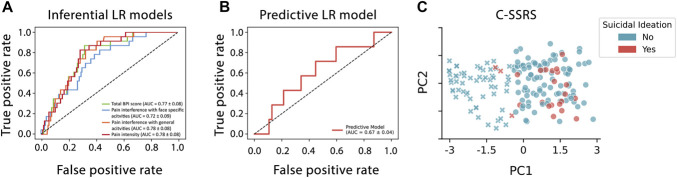
Analysis of the discriminative and predictive performance of the multivariable logistic regression models. (A) Each model incorporates 1 Brief Pain Inventory-Facial (BPI-Facial) scale domain (mean scores for pain intensity (red, AUC = 0.78 ± 0.08), interference with general activities (orange, AUC = 0.78 ± 0.08), or face-specific activities (blue, AUC 0.72 ± 0.09)) or the total BPI-Facial scale score (green, AUC = 0.77 ± 0.08) in predicting suicidal thoughts or behaviors. Models were adjusted for age, sex, and race. The dotted line represents chance-level performance. (B) The average, cross-validated receiver operating characteristic curve from the logistic-regression classifier model predicting suicidality in unseen data (AUC = 0.67 ± 0.04). The dotted line represents chance-level performance. (C) The 2 patient clusters identified using k-means methodology are grouped by whether patients exhibited suicidal ideation; ●, cluster A, ×, cluster B, red, suicidal ideation noted on Columbia-Suicide Severity Rating Scale (C-SSRS), blue, no suicidal ideation. LR, logistic regression; AUC, area under the curve; TN+, diagnosis of TN; PC1, principal component 1; PC2, principal component 2.

The discriminative performance of each model was assessed using the AUC (± standard error) of the ROC curve. The best performance was observed in the models based on pain severity (AUC = 0.78 ± 0.08) and interference with general activities (AUC = 0.78 ± 0.08), followed closely by total BPI-Facial scale score (AUC = 0.77 ± 0.08) and interference with face-specific activities (AUC = 0.72 ± 0.09; Fig. 2A).

To identify optimal cut-off scores for each BPI-Facial scale domain and the total BPI-Facial scale score in relation to suicidal thoughts or behaviors, YI was used to determine the threshold that maximized the sum of sensitivity and specificity for each predictor. For the pain intensity domain, the optimal cut-off score was >4.12, corresponding to a YI of 0.36, with a sensitivity of 91.3% and a specificity of 44.8%. For interference with general activities, the optimal threshold was >5.21 (YI = 0.41), yielding a sensitivity of 87.5% and specificity of 53.2%. For the total BPI-Facial scale score, the optimal cut-off was >118 (YI = 0.46), with a sensitivity of 75.0% and specificity of 71.1%. For interference with face-specific activities, the optimal score was >3.34 (YI = 0.26), with a sensitivity of 87.5% and specificity of 38.3%. These thresholds represent the points at which each scale component most effectively discriminates between individuals with and without suicidal thoughts or behaviors in unadjusted analyses. These cut-offs should not be interpreted as stand-alone screening thresholds but rather as indicators of the strength of the association between pain-related impairment and suicidal ideation.

The predictive logistic regression classifier model we developed exhibited modest cross-validated performance in identifying instances of suicidal ideation in unseen data (mean AUC ± SE = 0.67 ± 0.04) (Fig. 2B). Further analysis of the individual features driving the classification suggests that the model associated suicidal ideation with patients who had high pain intensity (β = 1.56) or were female (β = 1.44). Smaller contributions were observed with age (β = −0.70), interference in face-specific activities (β = −0.66), interference in general activities (β = 0.42), and having a diagnosis of TN (β = 0.03). Finally, the exploratory k-means methodology grouped patients into 2 distinct clusters (A and B). Patients in cluster A exhibited a greater average score across all domains compared with those in cluster B (pain intensity: 25.1 vs 8.3, interference in face-specific activities: 50.7 vs 12.6, interference in general activities: 45.8 vs 10.4). In addition, 87% of patients who exhibited suicidal ideation were assigned to cluster A (Fig. 2C).

## 4. Discussion

An understanding of the specific risks and predictors of suicidal ideation in patients with facial pain disorders is critical for the establishment of screening guidelines and targeted interventions. To this end, we performed a retrospective analysis of a large cohort of patients with facial pain disorders who were screened for suicidal ideation.

In line with previous studies, our analysis of BPI-Facial scale scores suggests that patients with pain disorders report moderate-to-high levels of pain intensity and multifaceted impairments in activities of daily living, ranging from impaired mood and sleep to difficulties with eating and speaking.^21^ The psychometric properties of the BPI-Facial scale, including its structure, reliability, and validity, have been previously reported.^10^ Subsequent research has suggested the following thresholds of improvement for minimum clinically important differences across BPI-Facial subscales: a 57% improvement in pain intensity at its worst, a 28% improvement in average pain, a 75% improvement with interference in activities of daily living, and a 62% improvement in interference in face-specific activities.^17^ We observed an elevated rate of suicidal ideation (13.3%) in this cohort, — approximately 3 times greater than estimates of suicidal ideation in the general adult population (∼4.3%).^16^ Critically, patients who reported suicidal ideation exhibited greater BPI-Facial scale scores across each of the domains tested (Fig. 1C).

Using multivariable logistic regression models, we identified robust associations between suicidal ideation and specific domains within the BPI-Facial scale, particularly regarding pain intensity and interference with general activities. These 2 domains were highly sensitive for suicidal ideation when using the optimal YI cutoff from the ROC curves (91.3% and 87.5% for pain intensity and interference with general activities, respectively). However, the models had lower specificity (44.8% and 53.2%, respectively), which suggests that scores in the pain intensity and interference with general activities domains have high negative predictive power (ruling out those who are unlikely to exhibit suicidal ideation) but may not be ideally suited for definitively identifying those at heightened risk of suicidal ideation, for whom other assessments may be more appropriate (eg, C-SSRS).

Next, to determine whether these same clinical and demographic features could predict instances of suicidal ideation or behaviors in unseen data, we used 2 different machine-learning techniques: a classification-based model and an exploratory cluster-based analysis. The application of machine-learning models can support suicide risk prediction and identify novel risk factors.^1^ In this vein, our results provided further support for the link between high BPI-Facial scale scores and suicidal ideation. Examination of model coefficients indicated that greater pain intensity and female sex carried the largest positive weights in classifying suicidal ideation, meaning these features contributed most strongly to model predictions rather than implying causal influence. Although our models were only trained on a few select features, we nonetheless achieved modest, above-chance performance in identifying instances of suicidal ideation (mean AUC = 0.67).

Incorporating standardized clinical questionnaires such as the C-SSRS and BPI-Facial scale into routine practice can provide a structured and scalable method to identify patients with facial pain who are at greater risk for suicide. These tools enable clinicians to quantify pain-related impairments and help uncover psychosocial stressors that may not emerge during standard clinical interviews. Our findings underscore the complementary value of integrating pain-specific assessments with standardized suicide-risk tools. The C-SSRS remains an appropriate screening instrument for identifying suicidal ideation, whereas the BPI-Facial scale can contextualize the extent of pain-related functional impairment that may exacerbate risk or guide individualized interventions. This is particularly relevant in neurosurgical practices, where management of patients with TN or other facial pain syndromes is common. Currently, there is no standardized protocol within neurosurgery for assessing mental health burden or suicide risk in this population. Our work highlights an unmet need and offers a foundation for the development of formalized screening and intervention protocols tailored to patients with facial pain disorders.

## 5. Limitations

Although the proportion of patients exhibiting suicidal ideation was greater than what is generally observed in the adult population, our ability to identify robust facial pain–related predictors of suicide risk was nonetheless limited by the small available sample. Therefore, aggregating patient data across institutions may facilitate more sophisticated modeling of clinical features via machine learning–based classification and clustering methods. In addition, our cohort comprised patients who participated in a presurgical evaluation, which represents a subset of the total population of patients with facial pain. Therefore, the patients in our cohort likely experienced more severe facial pain and debilitating impairments than those who received suitable symptom relief from pharmacological therapy. Although TN accounted for most cases in our cohort, we included other facial pain etiologies to capture the broader spectrum of pain-related impairment and associated psychosocial burden. This inclusive approach prioritized detection of generalizable associations across diagnoses rather than between-group comparisons.

The clinical questionnaires were administered only during the initial patient visit and not during subsequent follow-ups. Future research would benefit from a longitudinal characterization of suicidal risk and pain-related impairments to determine the differential effects of specific interventions, for instance, pharmacological management compared with surgical treatment. The retrospective design also precluded evaluation of additional factors known to influence suicide risk in pain populations, such as depression, anxiety, sleep disturbance, or pain catastrophizing. Consequently, our findings should be interpreted as preliminary and hypothesis generating.

Although we recorded the duration of symptoms, we did not evaluate symptom duration as an independent predictor of suicidal ideation. The median duration of symptoms (6.3 years), however, underscores the chronicity of pain in this cohort. Finally, corroboration of C-SSRS and BPI-Facial scale scores with validated clinical metrics (eg, Beck Depression Inventory, health-related quality of life) may further enrich our understanding of the detrimental effects of facial pain on mood and the activities of daily living.

Future studies should compare suicide risk across different chronic pain populations, including temporomandibular disorder and nonfacial pain syndromes, to determine whether observed associations are specific to facial pain.^5^ Prospective or interventional studies, such as randomized trials of psychotherapy or other risk mitigation interventions, could also clarify whether targeted treatment of pain-related impairment reduces suicidal ideation in these populations.

## 6. Conclusion

Patients with facial pain disorders exhibit diminished quality of life and a high incidence of comorbid psychiatric disease. In our cohort of patients with facial pain, we observed considerable pain-related impairments that were associated with suicidal ideation. Left unmanaged, these impairments exacerbate the burdens of a severely disabling disease. Comprehensive longitudinal screening and targeted interventions may be warranted to mitigate the heightened risk of suicide.

## Disclosures

J. D. Rolston reports consulting for NeuroPace, Turing, and AiM Robotics. The remaining authors have no conflicts of interest to declare.
